# Practical Surgery, with One Hundred and Twenty Engravings on Wood

**Published:** 1838-02-14

**Authors:** 


### BIBLIOGRAPHICAL NOTICES.

1. *Practical Surgery, with one hundred and twenty engravings on wood.* *By* Robert Liston, *Surgeon, London,* 1837. pp. 494. 8 vo.
2. *A Concise Treatise on Operative Surgery, describing the methods employed by the English, Continental, and American Surgeons; Selected for the use of Junior Practitioners and Students. Illustrated by twelve plates. By* Wm. P. Cocks, *Surgeon, &c.* pp. 375, 8vo.

We have no lack of good treatises in the operative department of Surgery. The works of Bell, Averill, Guthrie and others in England, and those of Roux, Sabatier, Coster, Tavernier, and the more recent one of Malgaigne, in France, it would be supposed were quite sufficient to give the young surgeon all the requisite knowledge in a branch of his art, which he should never have recourse to without regret. Mr. Liston's Essay will, we think, rank with any other production of the kind, and contrasts very favourably with his meagre and unsatisfactory "Elements," published some time since. He has brought to the task, requisites of a very high order, which eminently qualify him for its accomplishment. These are experience, judgment, and a well informed and well regulated mind. The style is attractive, easy, and concise. We perused the whole work indeed with much pleasure and instruction, and we cordially recommend it to the practitioner and student of Surgery. We may particularly indicate the chapters on "The Restoration of Lost Parts," and on "The Injuries and Diseases of the Genito-urinary apparatus," as being of especial value.

Our author's remarks upon the dressing of simple wounds appear to us judicious and well-timed. There can be but little doubt that in almost every instance an unnecessary excess of material is applied, heating the parts, annoying the patient, and retarding the healing process. Sir Astley Cooper entertains and promulgates the same views as Mr. Liston, and condemns unequivocally the common dressing of stumps after amputation.

His prejudices against unguents are we conceive in a great measure well grounded. The grease entering into their composition becomes, from the animal heat of the parts, rancid, and renders them an irritating as well as an unclean application. The medicinal properties of the articles employed may in general, we suspect, be more advantageously used in the form of lotions.

We cannot join Mr. Liston in his antipathy to poultices. Against these useful and comfortable applications he seems to possess an unconquerable horror and dread. He styles them "a filthy and uncomfortable application, from their weight and stench." "A poultice," says he, "the very word is disgusting by being associated with putrefaction and nastiness) has very seldom been employed in my hospital, or in my private practice, for the last ten or twelve years; in fact our nurses at the North London, have almost forgotten to make the abomination." p. 128.

As a substitute for the poultice, Mr. Liston uses the "water dressing," which consists of a double piece of lint of thick texture, soaked in hot water, and applied at an agreeable temperature; this is covered by oiled silk to prevent evaporation. "Heat and moisture, by which qualities a poultice produces its soothing and beneficial effects, by which the surface is relaxed, its capillary circulation encouraged, and discharge promoted, are thus simply afforded, without any of the weight, putrefaction, fermentation, stench and filth which is inseparable from the best and most scientifically contrived epithems and cataplasms." pp. 29—30.

The volume is illustrated with upwards of one hundred graphic and well executed engravings.

Mr. Cocks' Treatise appears to us rather as the respectable production of a tolerably expert compiler, than as possessing any valid claims to originality, or as the results of personal experience and practice. Mr. Cocks has however done the American Surgeon more justice than Mr. Liston, who with a solitary exception, is silent on the subject; yet we think his reading and knowledge of cisatlantic operations might be advantageously extended.

Among other things in this volume, we must confess that the following caption somewhat astonished and puzzled us at first sight. "*Case of Rhinoplastic operation for destruction of the lower lip.*" p. 164. Independent of the uncertainty excited at the obviously incorrect and involved phraseology of the whole sentence, which expresses directly the opposite meaning that we presume was intended to be conveyed, we were some time at a loss whether to rank the operation designated, as some novel and brilliant effort of the insular chirurgeon, or as a specimen of Mr. Cocks' lamentable crudity and ignorance, of which, did time permit us, we might furnish innumerable instances. We, in our simplicity, have hitherto always believed, that consonant with the derivation—ριν and πλασσω—the term rhinoplasty was limited to the reparation of the nasal feature, and that cheiloplasty referred to the new modelling of lips. Mr. Cocks, it would appear, is the parent of a new autoplastic nomenclature.

The lithographic delineations are neat and faithful.

